# Supplementary figures and images for: Cell-intrinsic vulnerability and immune activation cooperate to drive degeneration in a mitochondrial complex I deficiency model of optic neuropathy
Source: J Neuroinflammation. 2026 Feb 6;23:83. doi: 10.1186/s12974-026-03707-4 (PMC12977552; doi:10.1186/s12974-026-03707-4)

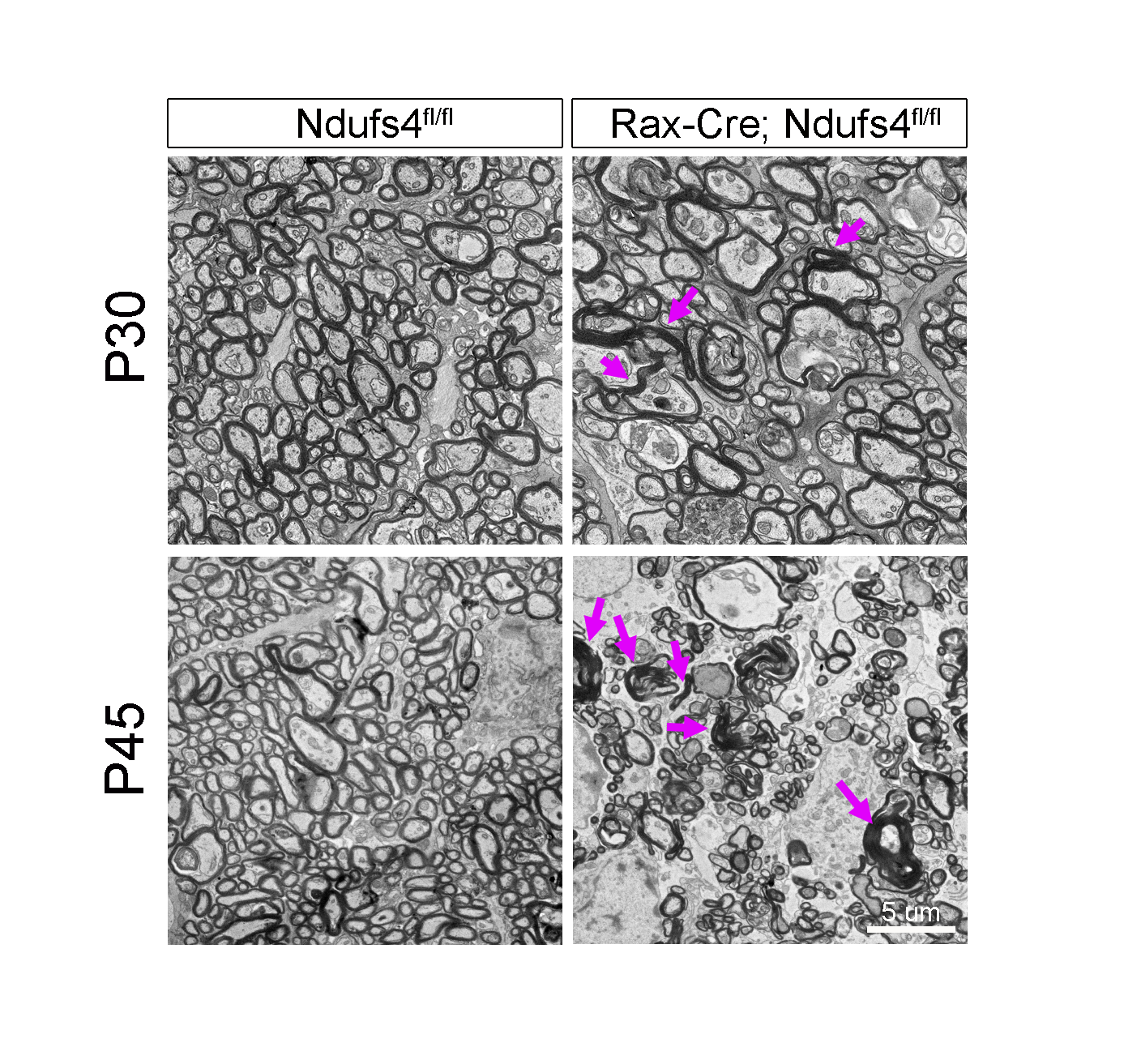

Supplement: Supplementary file 1 — Supplementary Material 1. Supplementary Fig S1. Ndufs4 loss results in defects of the myelin sheath. Electron microscopy images of retrobulbar optic nerves show disrupted myelin sheaths (arrows) in both P30 and P45 Rax-Cre; Ndufs4fl/fl animals. [file 12974_2026_3707_MOESM1_ESM.tif]

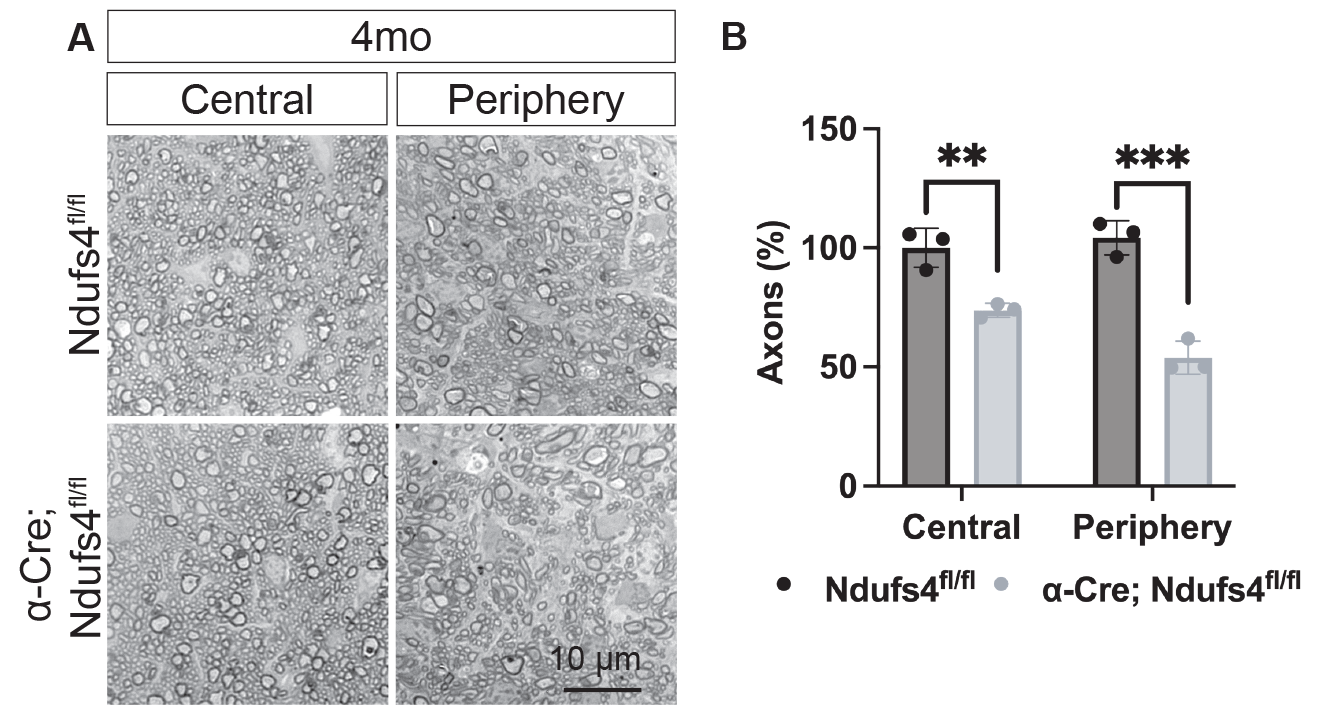

Supplement: Supplementary file 2 — Supplementary Material 2. Supplementary Fig S2. α-Cre; Ndufs4fl/fl animals show axonal degeneration at 4 months. A) Paraphenylenediamine (PPD) staining to label myelinated axons in optic nerve sections demonstrates axonal loss in α-Cre; Ndufs4fl/fl animals. Note that the degeneration is stronger in the periphery of the optic nerve. B) Quantification of the number of axons in PPD-stained optic nerve sections. Multiple t-tests, **P < 0.01 and ***P < 0.001). [file 12974_2026_3707_MOESM2_ESM.tif]

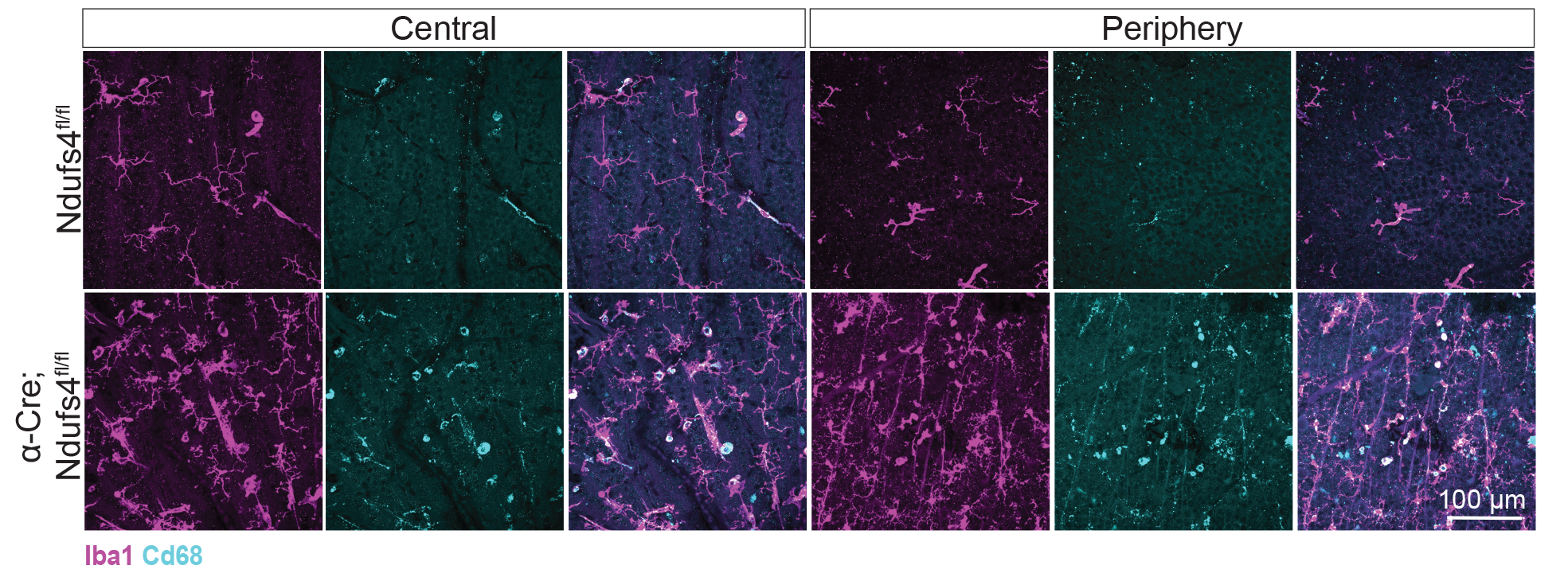

Supplement: Supplementary file 3 — Supplementary Material 3. Supplementary Fig S3. Activated myeloid cells are present in the center and periphery of α-Cre; Ndufs4fl/fl retinas. Retinal flat-mounts from P45 α-Cre; Ndufs4fl/fl mice immunostained for Iba1 (magenta) and Cd68 (teal) reveal a high density of activated myeloid cells across both central and peripheral regions of the GCL. Iba1⁺ cells co-express Cd68, a marker of activation and phagocytic activity, indicating widespread microglial activation in the degenerating retina. [file 12974_2026_3707_MOESM3_ESM.tif]

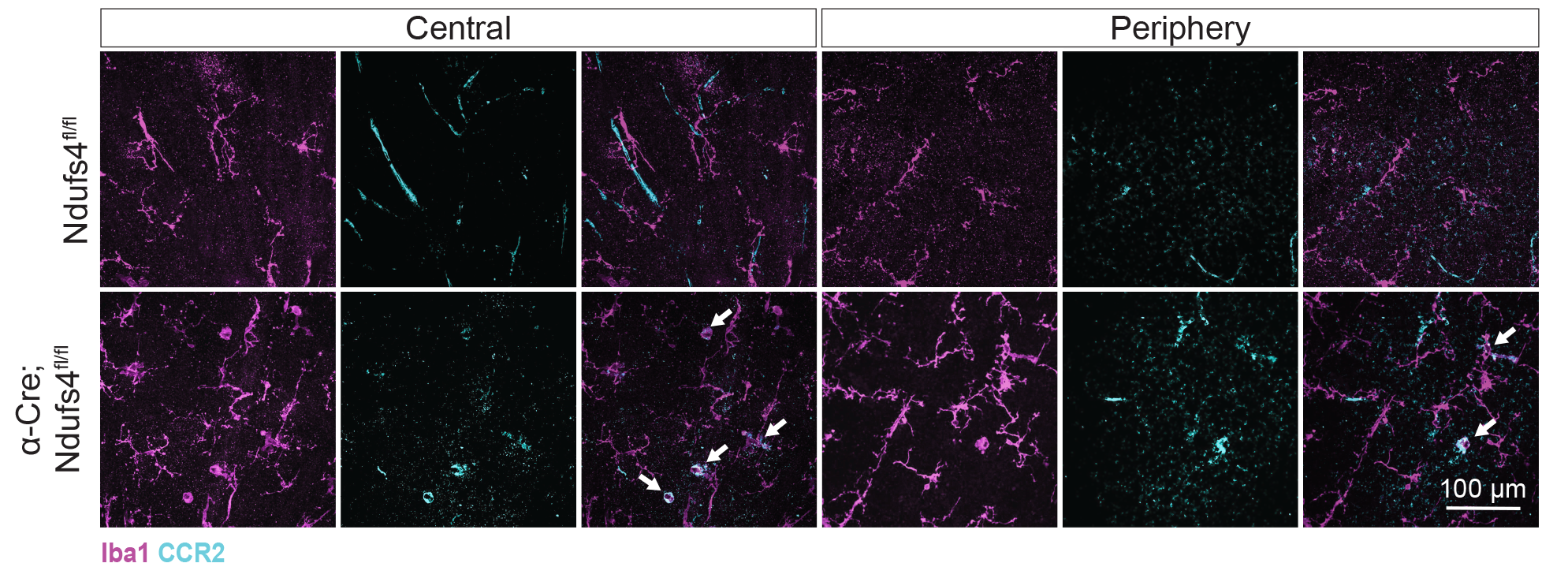

Supplement: Supplementary file 4 — Supplementary Material 4. Supplementary Fig S4. CCR2+ myeloid cells populate the retina of α-Cre; Ndufs4fl/fl mice at P45.Immunostaining of retinal flat-mounts from P45 α-Cre; Ndufs4fl/fl mice suggests the presence of infiltrating monocytes in both central and peripheral retina, indicated by co-localization of Iba1 (magenta) and CCR2 (teal) (arrows). [file 12974_2026_3707_MOESM4_ESM.tif]

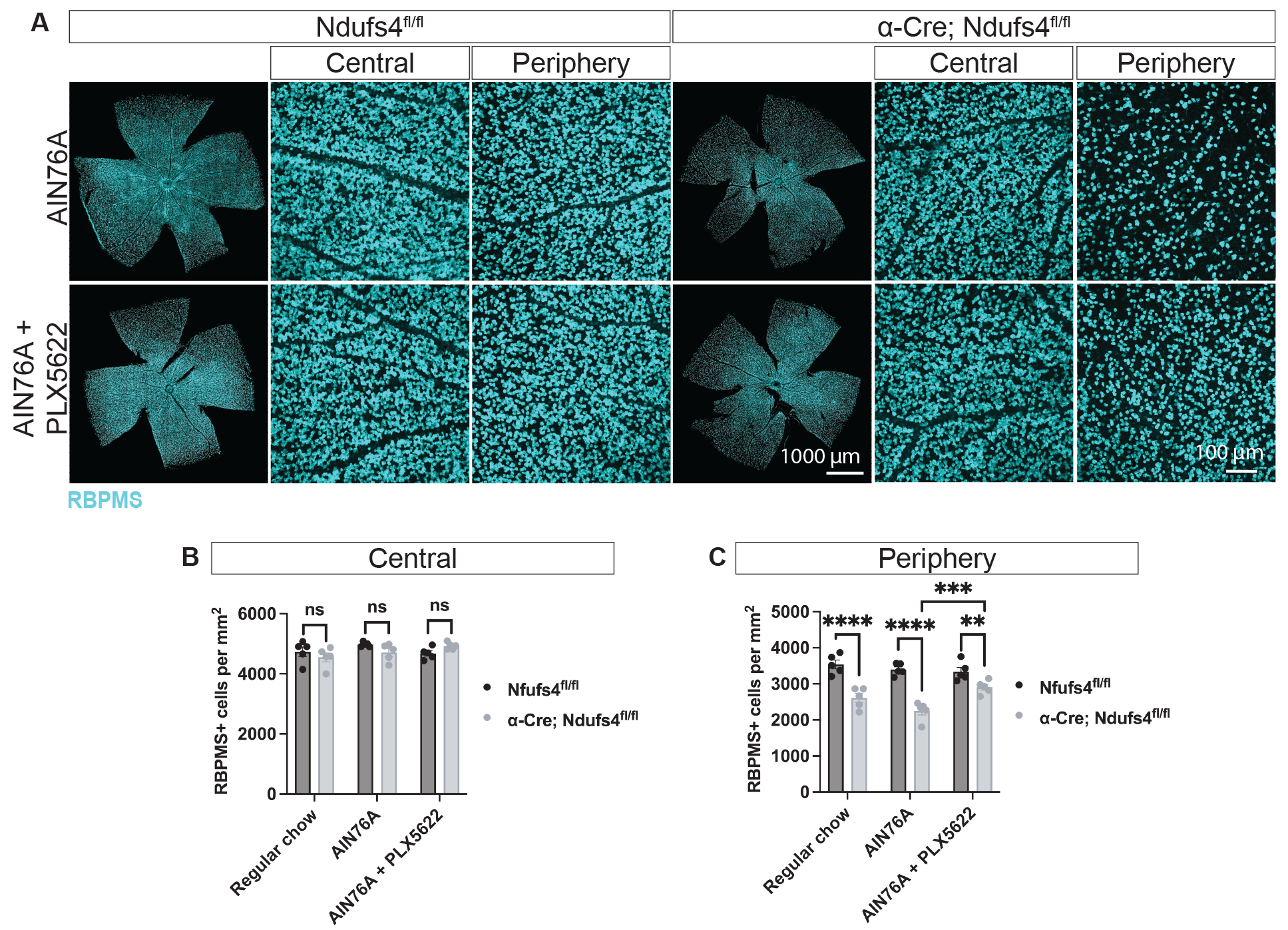

Supplement: Supplementary file 5 — Supplementary Material 5. Supplementary Fig S5. Myeloid cell depletion improves survival of peripheral RGCs in α-Cre; Ndufs4fl/fl retinas. A) Retinal flat-mounts stained with RBPMS (teal) show a partial rescue of RGCs in the periphery of α-Cre; Ndufs4fl/fl mice upon myeloid cell depletion with PLX5622 when compared to AIN76A control chow. B and C) Quantification of RBPMS+ RGCs per mm2 in animals fed with regular chow, AIN76A, or AIN76A supplemented with PLX5622. The central and peripheral retina were quantified separately (two-way ANOVA, n.s. not significant, **P < 0.01, ***P < 0.001, and ****P < 0.0001). [file 12974_2026_3707_MOESM5_ESM.tif]

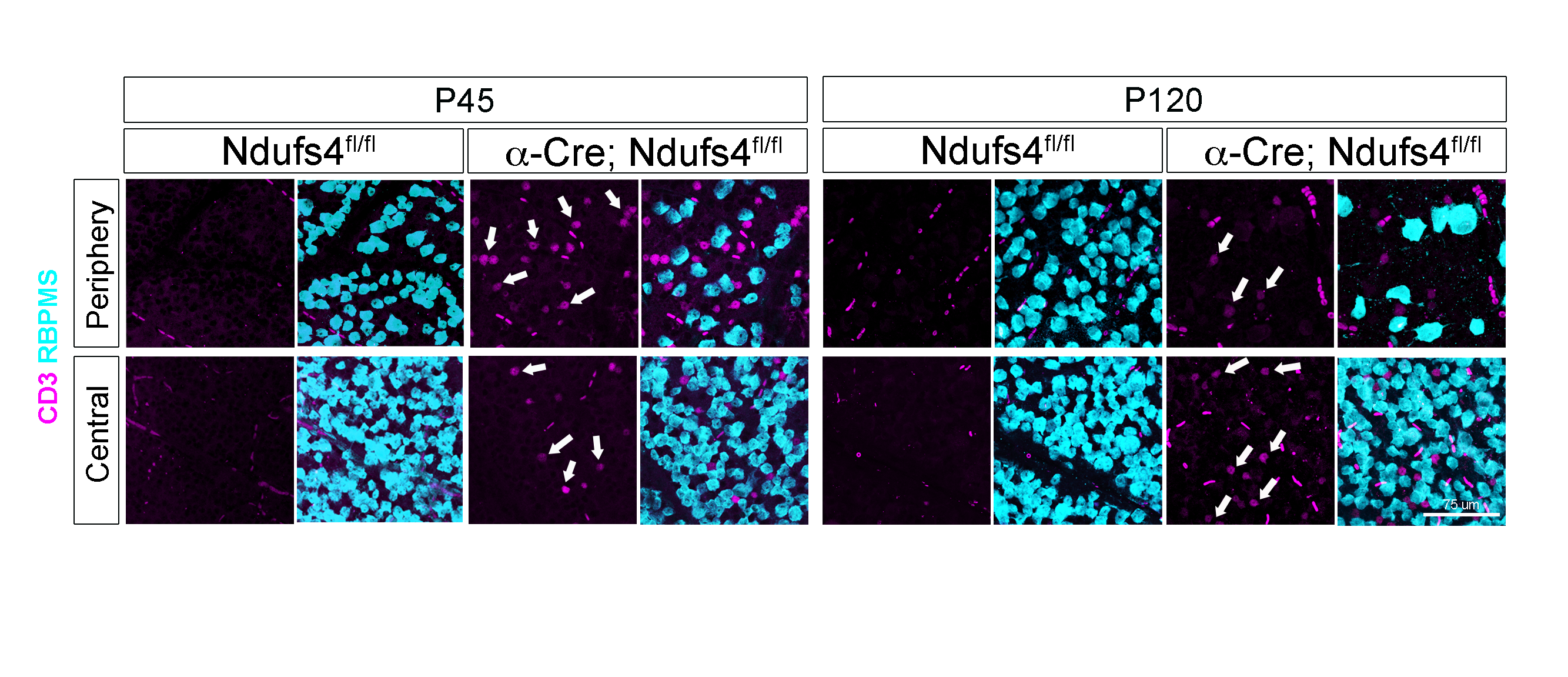

Supplement: Supplementary file 6 — Supplementary Material 6. Supplementary Fig S6. T-cells are present in α-Cre; Ndufs4fl/fl retinas. CD3 immunostaining (magenta) on flat-mounted retinas at P45 and P120 identified T-cells in both central and peripheral regions of α-Cre; Ndufs4fl/fl (arrows). Note the unspecific labeling of erythrocytes within blood vessels in all conditions. All samples were counterstained with RBPMS to identify RGCs. [file 12974_2026_3707_MOESM6_ESM.tif]
